# Safety of a single low-dose of primaquine in addition to standard artemether-lumefantrine regimen for treatment of acute uncomplicated *Plasmodium falciparum* malaria in Tanzania

**DOI:** 10.1186/s12936-016-1341-3

**Published:** 2016-06-10

**Authors:** Richard Mwaiswelo, Billy E. Ngasala, Irina Jovel, Roland Gosling, Zul Premji, Eugenie Poirot, Bruno P. Mmbando, Anders Björkman, Andreas Mårtensson

**Affiliations:** Department of Parasitology and Medical Entomology, Muhimbili University of Health and Allied Sciences, Dar es Salaam, Tanzania; Department of Microbiology, Tumor and Cell Biology, Karolinska Institutet, Stockholm, Sweden; Department of Epidemiology and Biostatistics, University of California San Francisco, San Francisco, CA USA; Global Health Group, University of California San Francisco, San Francisco, CA USA; Aga Khan University Hospital, Nairobi, Kenya; National Institute for Medical Research, Tanga Centre, Tanga, Tanzania; Department of Women’s and Children’s Health, International Maternal and Child Health (IMCH), Uppsala University, Uppsala, Sweden

**Keywords:** *Plasmodium falciparum* malaria, Primaquine, Glucose-6-phosphate dehydrogenase, Anaemia

## Abstract

**Background:**

This study assessed the safety of the new World Health Organization (WHO) recommendation of adding a single low-dose of primaquine (PQ) to standard artemisinin-based combination therapy (ACT), regardless of individual glucose-6-phosphate dehydrogenase (G6PD) status, for treatment of acute uncomplicated *Plasmodium falciparum* malaria in Tanzania.

**Methods:**

Men and non-pregnant, non-lactating women aged ≥1 year with uncomplicated *P. falciparum* malaria were enrolled and randomized to either standard artemether-lumefantrine (AL) regimen alone or with a 0.25 mg/kg single-dose of PQ. PQ was administered concomitantly with the first AL dose. All drug doses were supervised. Safety was evaluated between days 0 and 28. G6PD status was assessed using rapid test (CareStart™) and molecular genotyping. The primary endpoint was mean percentage relative reduction in haemoglobin (Hb) concentration (g/dL) between days 0 and 7 by genotypic G6PD status and treatment arm.

**Results:**

Overall, 220 patients, 110 per treatment arm, were enrolled, of whom 33/217 (15.2 %) were phenotypically G6PD deficient, whereas 15/110 (13.6 %) were genotypically hemizygous males, 5/110 (4.5 %) homozygous females and 22/110 (20 %) heterozygous females. Compared to genotypically G6PD wild-type/normal [6.8, 95 % confidence interval (CI) 4.67–8.96], only heterozygous patients in AL arm had significant reduction in day-7 mean relative Hb concentration (14.3, 95 % CI 7.02-21.55,* p*=0.045), however, none fulfilled the pre-defined haemolytic threshold value of ≥25 % Hb reduction. After adjustment for baseline parasitaemia, Hb, age and sex the mean relative Hb reduction was not statistically significant in both heterozygous and hemizygous/homozygous patients in both arms. A majority of the adverse events (AEs) were mild and unrelated to the study drugs. However, six (4.4 %) episodes, three per treatment arm, of acute haemolytic anaemia occurred between days 0 and 7. Three occurred in phenotypically G6PD deficient patients, two in AL and one in AL + PQ arm, but none in genotypically hemizygous/homozygous patients. All patients with acute haemolytic anaemia recovered without medical intervention.

**Conclusion:**

The findings support that the WHO recommendation of adding a single low-dose of PQ to standard AL regimen is safe for the treatment of acute uncomplicated *P. falciparum* malaria regardless of G6PD status in Tanzania.

*Trial registration number* NCT02090036

**Electronic supplementary material:**

The online version of this article (doi:10.1186/s12936-016-1341-3) contains supplementary material, which is available to authorized users.

## Background

Malaria prevalence is declining globally. The success is primarily accredited to the increased use of long-lasting insecticide treated bed-nets (LLIN) and artemisinin-based combination therapy (ACT) [1, 2]. This decline has rejuvenated dreams to eliminate malaria. However, to achieve this ambitious goal, new tools and strategies are needed, but equally important is to optimize the use of already available tools, such as malaria transmission-blocking drugs.

Primaquine (PQ) is an 8-aminoquinoline anti-malarial drug synthesized in 1945. It has been primarily used in combination with schizonticidal drugs to achieve radical *Plasmodium vivax* and *P. ovale* cure due to its effect on hypnozoites [3]. However, PQ also has an effect on mature *P. falciparum* gametocytes [4]. The main drawback of PQ use is dose-dependent acute haemolysis in glucose-6-phosphate dehydrogenase (G6PD) deficient individuals, a sex linked genetic disorder [5].

In 2012, the World Health Organization (WHO) issued a new recommendation to add a single low-dose of PQ (0.25 mg/kg) to standard ACT regimen regardless of G6PD status as a tool to interrupt transmission in low transmission settings and for containment in areas threatened by artemisinin resistance [6, 7]. However, the recommendation is based on low-quality evidence [6, 8, 9], and the potential impact of adding this single low-dose PQ to ACT on development of haemolysis following treatment for *P. falciparum* malaria is incompletely understood. Therefore, the aim of this study was to provide safety data on the new WHO recommendation when used in treatment of acute uncomplicated *P. falciparum* malaria in Tanzania, especially in patients with confirmed G6PD deficiency.

## Methods

### Study area

The trial was conducted at Yombo dispensary, Bagamoyo district, Tanzania between July and November 2014. The dispensary is located approximately 20 km from Bagamoyo town. It serves around 7000 people. The dispensary has laboratory capacity to carry out malaria microscopy and rapid diagnostic test. *P. falciparum* is the predominant malaria species and *Anopheles gambiae* sensu stricto the main vector [10, 11]. Artemether-lumefantrine (AL) is used as the first-line drug for uncomplicated malaria, and LLIN is the major vector control method. G6PD deficiency prevalence in the study area is estimated to be 9 % male hemizygous and 1.8 % female homozygous [12].

### Study design

This was a randomized, single-blinded clinical trial comparing safety and efficacy of AL versus a single low-dose PQ added to the standard AL treatment (AL + PQ). Clinical and parasitological cure rates will be presented in a separate publication. Patients with microscopically confirmed *P. falciparum* mono-infection were enrolled in the study, randomized to either AL or AL + PQ treatment, admitted during the first 3 days and thereafter followed-up until day-28 after treatment initiation.

### Study population

Participants were recruited from patients presenting at the study site with suspected acute uncomplicated *P. falciparum* malaria regardless of G6PD status. Inclusion criteria were: age ≥1 year, weight ≥10 kg, microscopy determined *P. falciparum* mono-infection, any parasitaemia level and a body temperature ≥37.5 °C or history of fever in the last 24 h, ability to swallow oral medication, ability and willingness to comply with the study protocol and stipulated follow-up visits, and written informed consent (in case of children a proxy consent from a parent/guardian).

Exclusion criteria included evidence of severe malaria or danger signs, known allergies to study medications, reported anti-malarial intake within the past 2 weeks, haemoglobin (Hb) concentration less than 8 g/dL, history of blood transfusion within last 90 days, febrile condition other than malaria, known underlying chronic or severe diseases, pregnancy and breastfeeding in the first 6 months.

### Randomization and blinding

Block randomization with four blocks, two per arm, was used for treatment assignment. The blocks were stratified by sex [13]. The randomization was performed using the computer software Research Randomizer version 4 (Wesleyan University, Connecticut, USA) [14]. Opaque envelopes containing the predetermined treatment codes were kept sequentially in a male and female strata. The envelopes were opened by a study nurse just prior to the administration of the first treatment dose. To achieve the single-blinding, the PQ dose was prepared in the absence of patients, mixed with a coloured glucose-based syrup and then administer. The coloured glucose-based syrup alone was administered with AL first dose to patients allocated AL alone.

### Treatment

All patients were treated with a standard three-day course of AL (Coartem^®^, Novartis) according to Tanzanian national treatment guidelines for uncomplicated *P. falciparum* malaria [15]. For patients allocated AL + PQ treatment, a single 0.25 mg/kg PQ dose (Primaquine phosphate, Sanofi) was prepared as previously described [13], and administered concomitantly with the first AL dose. All treatment doses were directly observed. A fatty snack (biscuits) was administered prior to all six AL doses to optimize absorption [16], and the single PQ dose to minimize gastrointestinal side effects [13]. All drug doses were administered by a study nurse, and the participants were observed for 30 min after each drug dose. Treatment was readministered in cases of vomiting within this time period.

### Patient withdrawals

Patients were withdrawn from the study in case of: vomiting the study drug over three times, withdrawal of consent, intake of any drug with anti-malarial properties outside the study protocol, or any protocol violation. Patients who missed a scheduled follow-up visit and would not be found despite efforts to trace them at their homes were considered lost to follow-up and consequently withdrawn. Patients with repetitive vomiting of study drug were managed according to national guidelines and followed up until recovery.

### Procedures

Clinical and laboratory assessments were performed on days 0, 1, 2, 3, 7, 10, 14, 21, 28, and on any day of recurrent illness. Every clinical assessment included history of clinical symptoms, possible adverse events, concomitant drug consumption and clinical examination including measurement of axillary temperature. All clinical and laboratory data were recorded in a case record form. The safety assessment was performed using the primaquine roll out monitoring pharmacovigilance tool (PROMPT) [17]. Adverse events (AE) and serious adverse event (SAE) were defined as previously defined [18].

At each visit, data on any post-treatment AE were collected with the following information recorded: type of AE (rash, nausea, vomiting, anaemia, dark urine, other), date of onset, severity grade (mild, moderate, severe), any actions taken (prescribed drug, blood transfusion, other), its relatedness to PQ (definitely related, probably, possibly, unlikely, not related), date resolved, outcome of AE, and any concomitant drug intake during the study.

All patients received an information card with instructions on how to identify signs and symptoms of commonly reported AEs including monitoring of urine colour. In addition, patients were given a clear container and asked to provide a urine sample on day 0 prior to first drug-dose and on days 1 and/or 2 or 3. The urine colour estimation of haemoglobinuria was gauged against the Hillmen colour chart with a colour score ranging from 1 to 10 [19]. Staff assigning colours to urine were blinded to the treatment allocation. A colour score ≥5 was considered evidence of haemoglobinuria indicating acute haemolysis. Acute haemolysis was defined as fractional Hb fall ≥25 % from the pre-treatment value or macroscopic haemoglobinuria (Hillmen ≥5) between the day of enrolment and day 7. Acute haemolytic anaemia was defined as any Hb drop to ≤7 g/dL, or ≥25 % from the pre-treatment value accompanied by a Hillmen urine colour score ≥5 between the day of enrolment and day 7.

Laboratory assessment involved collection of finger-prick blood samples for Hb concentration, thick film for microscopy to assess presence and density of asexual parasitaemia and gametocytaemia, and thin film for species determination.

Hb concentration was measured using a portable spectrophotometer, HemoCue Hb 201 + (HemoCue AB, Ängelholm Sweden), with a precision of ±0.3 g/dL. The HemoCue was calibrated every morning by using a control cuvette at 16.0 ± 0.3 g/dL according to manufacturer’s instruction.

The smears were stained using 10 % Giemsa and read by qualified microscopists. Asexual parasites on thick smears were counted against 200 white blood cells (WBC). This number was multiplied by 40, assuming 8000 leukocytes per microlitre of blood, to gain an approximate parasite density. A blood slide was considered negative if no parasites were seen after examining 100 fields. Two, independent microscopists read all microscopy slides. In cases of discordance in the presence of parasitaemia, or in parasite density by more than 25 %, a third independent reading was done. The majority result was taken as final in case of positive versus negative results and the geometric mean of the two closest parasite density estimates.

At enrolment, phenotypic G6PD screening and filter paper blood sampling for polymerase chain reaction (PCR)-based G6PD genotyping were also collected using capillary blood. The filter papers (3MM Whatman) were labeled, air-dried at room temperature for 3–4 h, packed in individual plastic bags, and then transported to Karolinska Institutet, Sweden, for molecular analysis.

G6PD phenotypic screening was performed at inclusion using a rapid enzyme chromatographic test (CareStart™, Access Bio, Inc. New Jersey, USA), (Cat. Ref. No. G0221), following the manufacturer instructions. Test results were read visually after 10–15 min. A distinct purple colour in the result window was interpreted as normal G6PD activity, while no colour change or a very faint purple colour was interpreted as deficient G6PD activity. The quality of rapid test reading was controlled by two or more individuals reading the test. The test was repeated in case the research team is not satisfied with the test result.

G6PD genotyping was performed from dried blood spots on filter paper. Genomic DNA was extracted using 10 % chelex [20], followed by DNA amplification using single-round PCR and restricted fragment length polymorphism (RFLP) digestion using restriction enzymes for the two most common polymorphisms associated with G6PD deficiency in Africa (i.e., G6PD A376G and G202A), as previously described [12]. The digested products were loaded on 3 % agarose gel containing GelRed™ (Biotium, Inc. Hayward, California, USA), separated by electrophoresis and visualized in a Gel Doc™ (Bio-Rad, Hercules, California, USA) under ultraviolet light using Image Lab™ software (Bio-Rad, Hercules, California, USA). First, G6PD A376G analysis was performed. All samples with 376G mutation were then subjected to G6PD G202A analysis and the outcomes were classified as follows: for males A was defined as wild-type/normal and A- as hemizygous/deficient G6PD status, whereas for females A-A- was defined as homozygous/deficient, AA- and BA- as heterozygous/intermediate, and AA and BA as wild-type/normal G6PD status [12].

### Study end-points

The primary endpoint was mean relative reduction in Hb concentration (g/dL) between day 0 and 7. Secondary endpoints included: mean absolute reduction in Hb concentration between days 0 and 7; proportion of patients with a Hillmen urine colour score ≥5 between days 1 and 3; proportion of patients with adverse and serious adverse events; and the proportion of patients phenotypically/genotypically G6PD deficient.

### Ethical considerations

The study was conducted in accordance with Good Clinical Practices (GCP), the Declaration of Helsinki, and applicable regulatory requirements in Tanzania. The trial was approved by the Muhimbili University of Health and Allied Sciences and the National Institute for Medical Research ethics committees. Importation and use of PQ for research purposes were approved by the Tanzania Food and Drug Authority. The Committee on Human Research at the University of California San Francisco approved the use of PROMPT to sites interested in its use. An independent data monitoring and safety board conducted an interim safety analysis during the trial. Written informed consent was obtained from all patients and a proxy consent from parents/guardians in patients aged <18 years, prior to enrolment. The study is registered at clinicaltrials.gov (NCT02090036).

### Statistical analysis

A total sample of 220, 110 per treatment arm, was calculated to be sufficient to detect within person reduction in Hb of ≥25 % between days 0 and 7 with 80 % power and a significance level of 0.05 (one-sided), assuming a mean Hb of 10 g/dL, a standard deviation of 2 and 25 % loss to follow-up. This sample size is also sufficient to detect the reduction in Hb of ≥25 % in, a population with genotypic G6PD deficiency prevalence of 10 %.

Data were double entered in an electronic database and analysed using SPSS software version 16 (SPSS Inc, Chicago, USA). All the assessments were done by phenotypic/genotypic G6PD status and treatment arm. Independent sample *t* test was used to compare absolute and relative mean changes in Hb concentration in hemizygous/homozygous and heterozygous patients against G6PD wild-type/normal, or phenotypic G6PD deficient against normal patients within treatment arms. In an additional assessment, phenotypic or genotypic (hemizygous/homozygous) G6PD deficient patients from both arms were pooled, and their absolute and relative mean changes in Hb concentration were compared against G6PD normal patients. Pre-treatment Hb, parasitaemia, age and sex were included in a linear regression model to adjust for their effect on relative Hb reduction between days 0 and 7. Comparison of proportions was performed using *Chi* square test. *Kappa* test was used to test the agreement between phenotypic and genotypic G6PD test results. Data from patients lost to follow-up or who withdrew consent were censored at the time of exit from study. A *p* < 0.05 was defined as statistically significant.

## Results

**Fig. 1:**
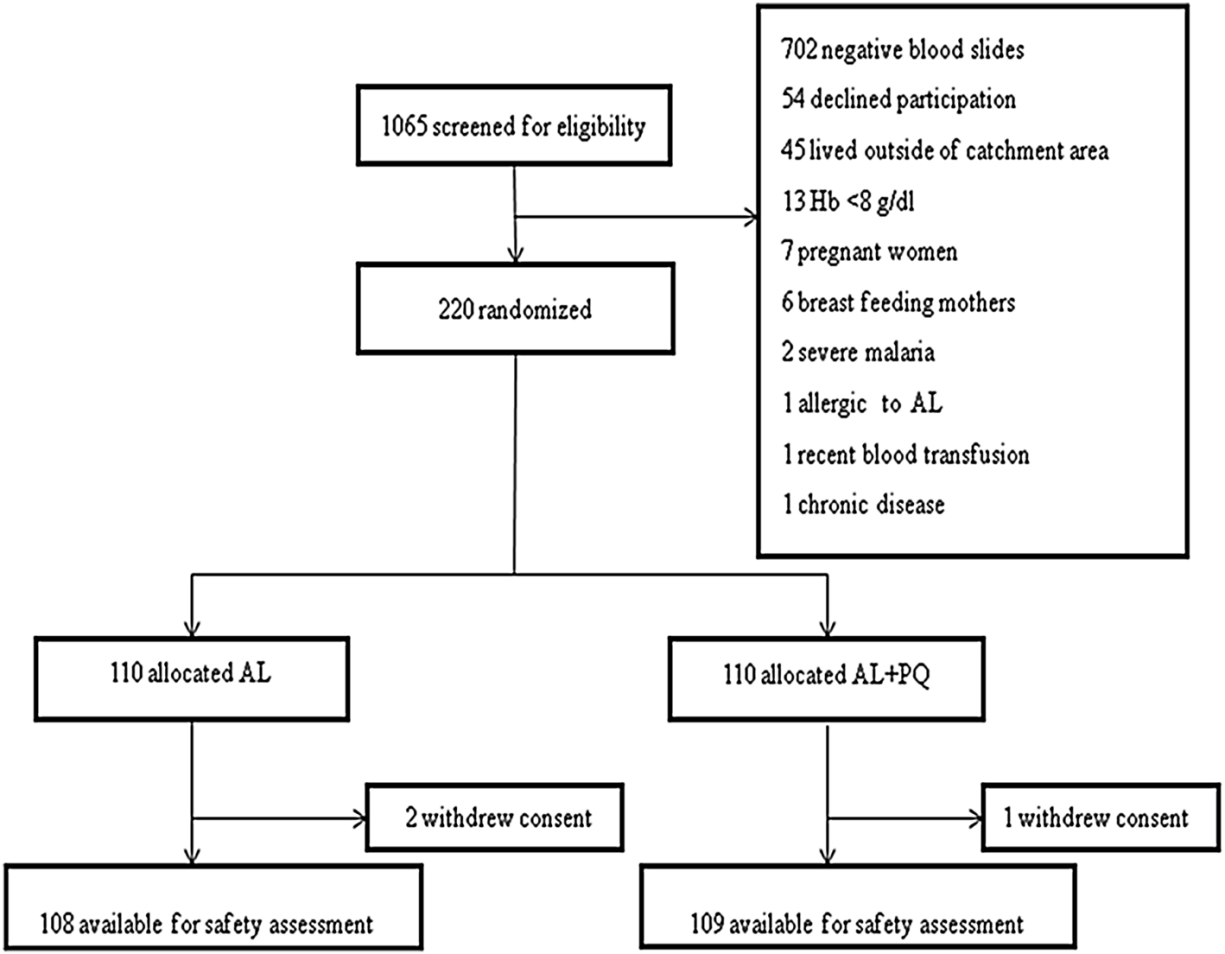
Trial profile of the study participants

A total of 1065 patients was screened for eligibility, 363 (34.1 %) had a positive *P. falciparum* blood slide, of whom 143 were excluded (Fig. 1). The remaining 220 were enrolled in the study, 110 each were allocated AL and AL + PQ treatment. Baseline characteristics of the study groups are presented in Table 1.

**Table 1:** Baseline characteristics

Characteristic	Treatment arm		*P* value
	AL (N = 110)	AL + PQ (N = 110)	
Female sex, n (%)	55 (50)	55 (50)	
Age (years), median (range)	10 (1–84)	9 (1–75)	0.392^a^
Body weight(kg), mean (SD)	34.3 (19.6)	30.7 (17.1)	0.145^b^
Body temperature(^0^C), mean (SD)	38.3 (1.3)	38.3 (1.1)	0.977^b^
Haemoglobin concentration (g/dl), mean (SD)	11.4 (1.6)	11.1 (1.5)	0.097^b^
Geometric mean asexual parasite density/µl, (range)	8384 (240–389,045)	8327 (240–288,403)	0.982^b^
Fever (> 37.5 °C) at inclusion, n (%)	80 (72.7)	88 (80.0)	0.204^c^

^a^ Mann–Whitney test

^b^Student t-test

^c^ *Chi* square test

### G6PD status

#### PhenotypicG6PD status

Phenotypic G6PD results were available for 217 (98.6 %) patients, 33 (15.2 %) were G6PD deficient, of whom 21/107 (19.6 %) were males and 12/110 (10.9 %) females. The distribution of phenotypic G6PD deficiency was 19/109 (17.4 %) in the AL arm and 14/108 (12.9 %) in the AL + PQ arm, (*p* = 0.36).

#### Genotypic G6PD status

**Fig. 2:**
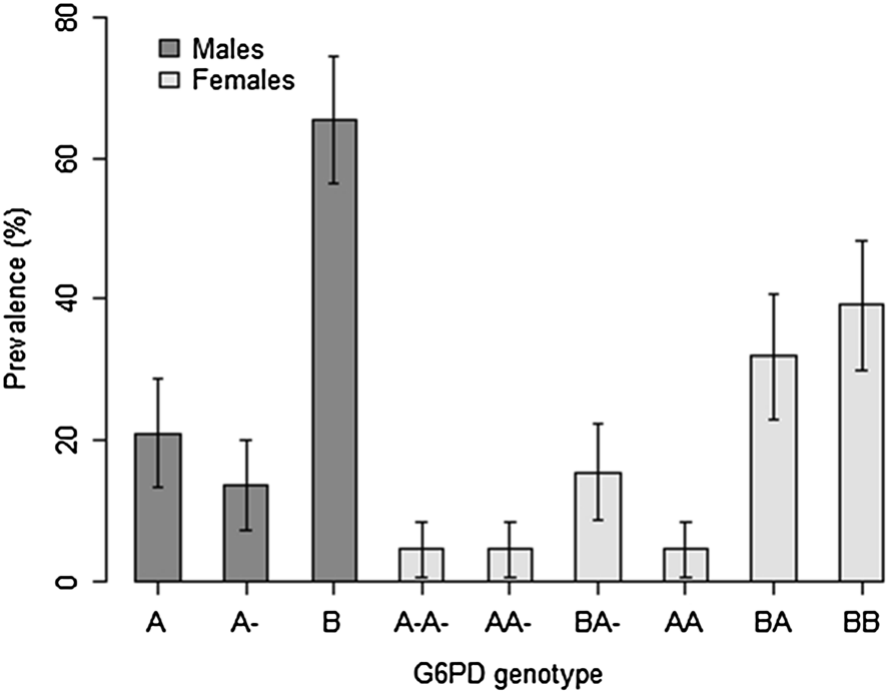
G6PD G202A allele frequency distribution. (Male hemizygous = A-; Male wild-type/normal = A or B; Female homozygous = A-A-; Female heterozygous = AA- or BA-; Female wild-type/normal = AA, BA or BB)

G6PD allele frequency distribution is presented in Fig. 2, whereas distribution of patients by sex, genotypic G6PD status and treatment arm are presented in Table 2. Stratification of patients by sex and genotypic G6PD status did not affect distribution of patients within treatment arms.

**Table 2:** Distribution of patients by sex, genotypic G6PD status and treatment arm

Treatment arm	Sex				
	Male		Female		
	Hemizygous	Wild-type/normal	Homozygous	Heterozygous	Wild-type/normal
AL	10 (18.2 %)	45 (81.8 %)	1 (1.8 %)	10 (18.2 %)	44 (80.0 %)
AL + PQ	5 (9.1 %)	50 (90.9 %)	4 (7.3 %)	12 (21.8 %)	39 (70.9 %)
Total	15 (13.6 %)	95 (86.4 %)	5 (4.5 %)	22 (20.0 %)	83 (75.5 %)

#### Phenotypic and genotypic G6PD test agreement

Overall agreement between phenotypic and genotypic G6PD test results was poor (*Kappa coefficient* 0.076, *p* = 0.29). The agreement between the tests for individual G6PD genotypes is presented in Table 3.

**Table 3:** Relationship between phenotypic and genotypic G6PD test results

Genotype results	Phenotype results	N/N (%)
Male hemizygous	Deficient	9/14 (64.3)
	Normal	5/14 (35.7)
Male wild-type/normal	Deficient	12/93 (12.9)
	Normal	81/93 (87.1)
Female homozygous	Deficient	0/5 (0)
	Normal	5/5 (100)
Female heterozygous	Deficient	4/22 (18.2)
	Normal	18/22 (81.8)
Female wild-type/normal	Deficient	8/83 (9.6)
	Normal	75/83 (90.4)

### Hb reduction between days 0 and 7 by phenotypic or genotypic G6PD status and treatment arm

#### Absolute Hb reduction

The absolute mean Hb reduction by phenotypic G6PD status is presented in Table 4a. Pooling all 33 phenotypic G6PD deficient patients together did not result in any statistically significant difference in Hb reduction compared with G6PD normal patients.

**Table 4:** Mean Hb reduction between days 0 and 7 by G6PD status and treatment arm

Characteristic	Treatment arm	
	AL	AL + PQ
A: Mean absolute and relative Hb reduction by phenotypic G6PD status		
*Mean absolute Hb reduction (g/dL) (95* *% CI)*		
G6PD normal	0.83 (0.57–1.10)	0.81 (0.59–1.02)
G6PD deficient	0.88 (0.32–1.45)	1.16 (0.67–1.66)
*P* value	0.87	0.23
*Mean relative Hb reduction (%) (95* *% CI)*		
G6PD normal	6.4 (4.29–8.56)	7.2 (5.04–9.35)
G6PD deficient	7.8 (4.23–11.36)	7.9 (4.58–11.18)
*P* value	0.71	0.16
B: Mean absolute and relative Hb reduction by genotypic G6PD status		
*Mean absolute Hb reduction (g/dL) (95* *% CI)*		
G6PD wild-type/normal	0.82 (0.56–1.1)	0.74 (0.52–0.95)
G6PD heterozygous	1.68 (0.87–2.51)	1.27 (0.56–1.99)
*P* value	0.048	0.09
G6PD hemizygous/homozygous	0.35 (−0.36–1.1)	1.48 (0.59–2.37)
P value	0.21	0.047
*Mean relative Hb reduction (%) (95* *% CI)*		
G6PD wild-type/normal	6.8 (4.67–8.96)	6.2 (4.25–8.17)
G6PD heterozygous	14.3 (7.02–21.55)	10.9 (5.10–16.87)
*P* value	0.045	0.09
G6PD hemizygous/homozygous	2.7 (4.22–9.68)	12.6 (6.0–19.25)
*P* value	0.21	0.059

The absolute mean Hb reduction by genotypic G6PD status is presented in Table 4b. The absolute mean Hb reduction among heterozygous and hemizygous/homozygous patients treated with AL was 1.68 g/dL (95 % CI 0.87–2.51), *p* = 0.048, and 0.35 g/dL (95 % CI 0.36–1.1), respectively. The corresponding results in the AL + PQ arm were, 1.27 g/dL (95 % CI 0.56–1.99, *p* = 0.09) and 1.48 g/dL (95 % CI 0.59–2.37, *p* = 0.047), respectively. After all 20 hemizygous/homozygous patients were pooled and compared with G6PD wild-type/normal, no statistically significant difference in Hb reduction was observed.

**Fig. 3:**
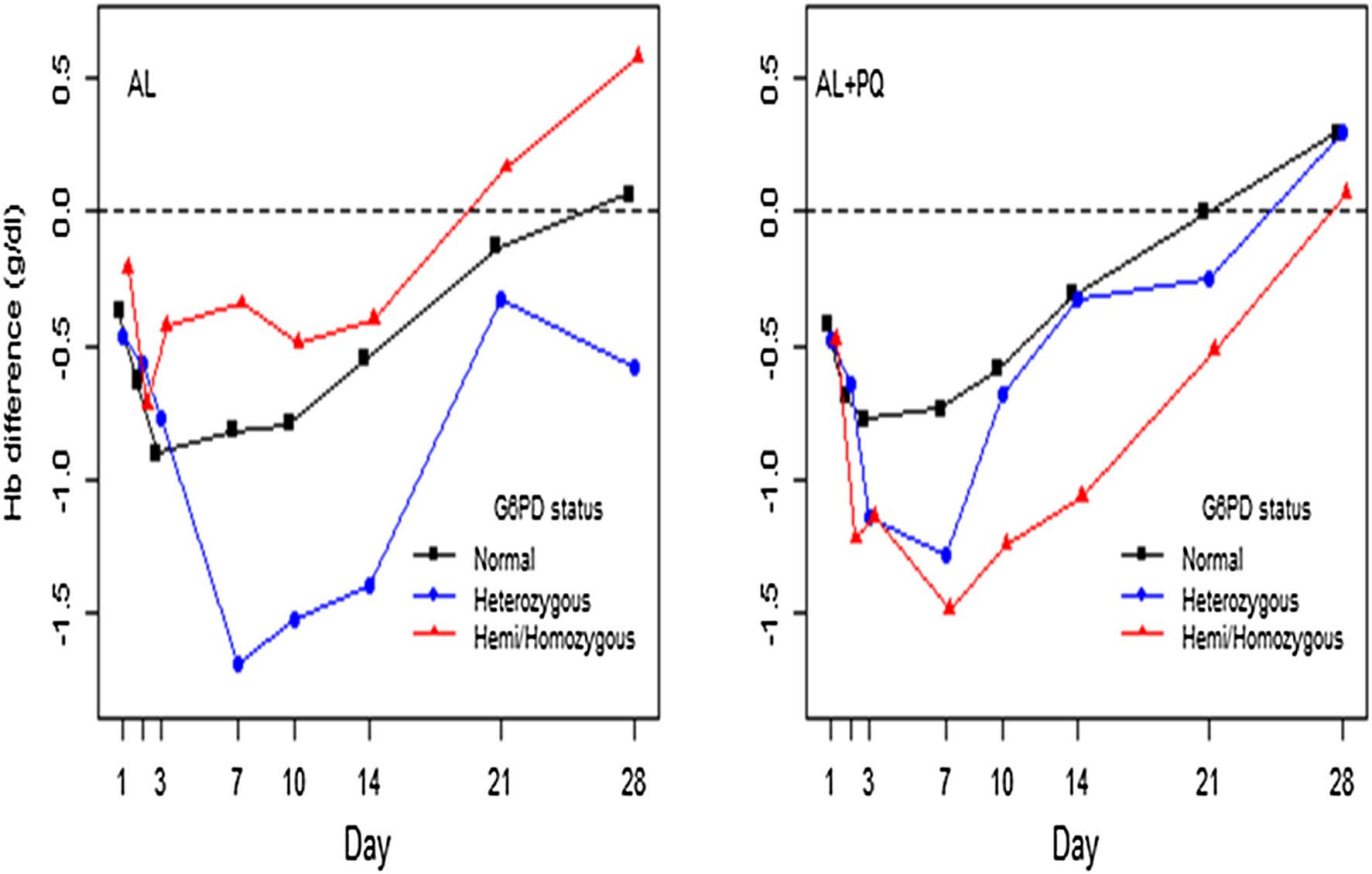
Mean absolute change in Hb concentration (g/dl) per treatment arm and genotypic G6PD status. (*Normal* represents wild-type)

A total of 54/101(53.5 %) and 60/105 (57.1 %), *p* = 0.59, patients treated with AL and AL + PQ arms, respectively, recovered their Hb by day 28 to or above the initial values (Fig. 3). After the phenotypic/genotypic deficient patients were pooled, there was no statistically significant difference in the proportion of patients with Hb recovery by day 28 compared with G6PD normal patients.

#### Relative Hb reduction

The percentage relative mean Hb reductions by phenotypic G6PD status are presented in Table 4a. A pooling of all phenotypically G6PD deficient patients did not result in any statistically significant difference in Hb reduction compared with G6PD normal patients.

The mean Hb reduction by genotypic G6PD status are presented in Table 4b. The relative mean Hb reduction in heterozygous patients treated with AL was 14.3 % (95 % CI 7.02–21.55, *p* = 0.045). The mean Hb reduction in heterozygous and hemizygous/homozygous patients treated with AL + PQ did not differ significantly to G6PD wild-type/normal patients. The percentage relative Hb reduction after adjustment for baseline parasitaemia, Hb, age and sex are presented in Table 5. After adjusting for the baseline characteristics, the relative Hb reduction was non-significant in both heterozygous and hemizygous patients. Pooling hemizygous/homozygous patients together did not result in any statistically significant difference in Hb reduction compared with G6PD wild-type/normal.

**Table 5:** Linear regression models of relative change in Hb between days 0 and 7

Treatment arm	Variable	Coefficient	95 % CI	*P* value
AL	Heterozygous	−6.61	−13.75 to 0.53	0.124
	Hemi/Homozygous	4.29	−1.87 to 10.45	0.407
AL + PQ	Heterozygous	−3.97	−8.98 to 1.03	0.095
	Hemi/Homozygous	−6.22	−12.22 to −0.22	0.068

Adjustment was done for baseline Hb, parasite density, age and sex

**Fig. 4:**
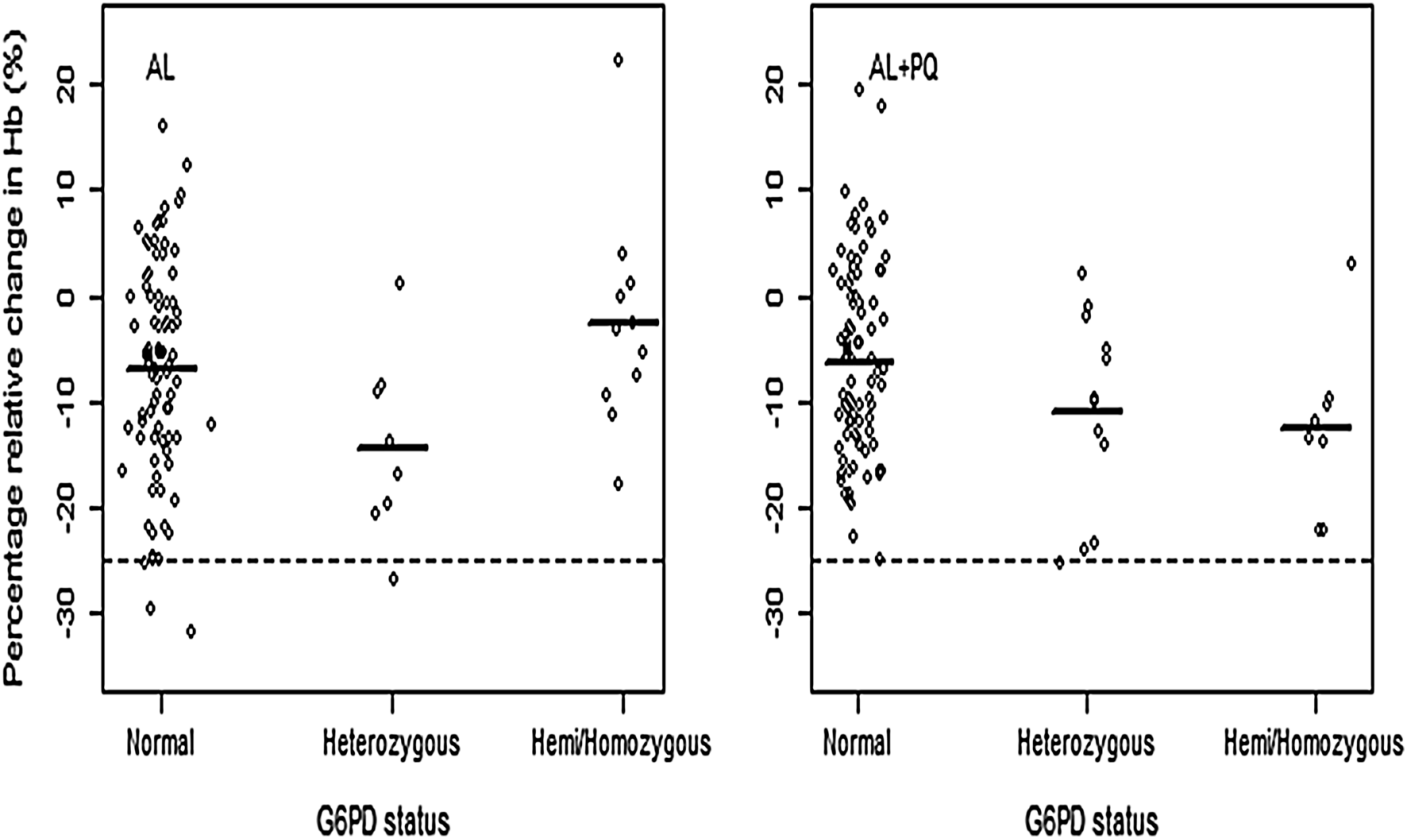
Mean relative change in Hb concentration (g/dl) per treatment arm between days 0 and 7. (*Points* show individual relative change, solid line segment is the mean while the *dotted line* indicates a threshold line at 25 %. *Normal* represents wild-type)

A total of 4/89 (4.5 %) and 1/10 (10.0 %) G6PD wild-type/normal and heterozygous patients, respectively, treated with AL experienced an Hb reduction ≥25 %. The corresponding results in the AL + PQ arm were 1/89 (1.1 %) and 1/12 (8.3 %), respectively. None of the hemizygous/homozygous patients in either the AL or AL + PQ arm fulfilled the pre-defined haemolytic threshold (Fig. 4). Some 1/19 (5.3 %) and 4/86 (4.7 %) of phenotypic G6PD deficient and normal patients, respectively, treated with AL had an Hb reduction ≥25 %. However, none of the G6PD deficient and 2/107 (1.9 %) normal patients in AL + PQ arm had an Hb reduction ≥25 %.

### Adverse events

The distribution of AEs across phenotypic and genotypic G6PD status by treatment arm is presented in Table 6. AEs occurred in 41.8 % (92/220) of patients, 42.7 % (47/110) in AL + PQ and 40.9 % (45/110) in AL arm, *p* = 0.79. Patients with haemoglobinuria in AL + PQ arm had significantly higher mean pre-treatment parasite density. Most AEs were mild (grade 1) and considered unrelated to the study drugs. However, six (4.4 %) episodes of acute haemolytic anaemia, three in each treatment arm, occurred (Additional file 1). Despite fulfilling the pre-defined definition of acute haemolytic anaemia all were in overall good general condition and did not require any medical intervention. There was no statistically significant association between baseline characteristics and reported AE.

**Table 6:** Distribution of adverse events by phenotypic or genotypic G6PD status and treatment arm

Adverse event	Treatment arm					
	AL		AL + PQ			
	Normal	Deficient	Normal	Deficient		
*A: Distribution of adverse events by phenotypic G6PD status*						
Abdominal pain	6 (10.5 %)	1 (7.1 %)	6 (10.7 %)	1 (11.1 %)		
Blurred vision	1 (1.8 %)	0	0	0		
Cold sores	2 (3.5 %)	1 (7.1 %)	4 (7.1 %)	0		
Diarrhoea	3 (5.3 %)	1 (7.1 %)	2 (3.6 %)	1 (11.1 %)		
Dizziness	2 (3.5 %)	0	1 (1.8 %)	0		
Fever	21 (36.8 %)	3 (21.4 %)	14 (25.0 %)	2 (22.2 %)		
Headache	7 (12.3 %)	1 (7.1 %)	5 (8.9 %)	0		
Hb fall ≥ 25 %	5 (8.8 %)	2 (14.3 %)	5 (8.9 %)	0		
Acute haemolytic anaemia	1 (1.8 %)	2 (14.3 %)	2 (3.6 %)	1 (11.1 %)		
Haemoglobinuria	1 (1.8 %)	2 (14.3 %)	10 (17.9 %)	4 (44.4 %)		
Nausea	2 (3.5 %)	0	0	0		
Rashes	0	0	1 (1.8 %)	0		
Vomiting	5 (8.8 %)	1 (7.1 %)	6 (10.7 %)	0		
Weakness	1 (1.8 %)	0	0	0		
Total	57 (100 %)	14 (100 %)	56 (100 %)	9 (100 %)		

	AL			AL + PQ		
	Wild-type/normal	Heterozygous	Hemizygous/homozygous	Wild-type/normal	Heterozygous	Hemizygous/homozygous
*B: Distribution of adverse events by genotypic G6PD status*						
Abdominal pain	7 (11.3 %)	0	0	4 (8.0 %)	4 (44.0 %)	0
Blurred vision	1 (1.6 %)	0	0	0	0	0
Cold sores	3 (4.8 %)	0	0	4 (8.0 %)	0	0
Diarrhoea	2 (3.2 %)	1 (12.5 %)	1 (100 %)	3 (6.0 %)	0	0
Dizziness	1 (1.6 %)	1 (12.5 %)	0	0	1 (10.0 %)	0
Fever	21(33.9 %)	3 (37.5 %)	0	10 (20.0 %)	3 (30.0 %)	3 (50.0 %)
Headache	8 (12.9 %)	0	0	4 (8.0 %)	0	1 (16.7 %)
Hb fall ≥ 25 %	5 (8.1 %)	2 (25.0 %)	0	5 (10.0 %)	0	0
Acute haemolytic anaemia	3 (4.8 %)	0	0	2 (4.0 %)	1 (10.0 %)	0
Haemoglobinuria	3 (4.8 %)	0	0	12 (24.0 %)	1 (10.0 %)	1 (16.7 %)
Nausea	1 (1.6 %)	1 (12.5 %)	0	0	0	0
Rashes	0	0	0	1 (2.0 %)	0	0
Vomiting	6 (9.7 %)	0	0	5 (10.0 %)	0	1 (16.7 %)
Weakness	1 (1.6 %)	0	0	0	0	0
Total	62 (100 %)	8 (100 %)	1 (100 %)	50 (100 %)	10 (100 %)	6 (100 %)

## Discussion

The results from this randomized clinical trial supports the view that the new WHO recommendation of adding a single low-dose of PQ to standard AL regimen was safe for the treatment of acute uncomplicated *P. falciparum* malaria in Tanzania regardless of individual G6PD status.

In this study, only G6PD heterozygous patients treated with AL had statistically significant percentage relative Hb reduction, but none fulfilled the ≥25 % haemolytic threshold. After adjustment for baseline characteristics, none of the patient had significant Hb reduction either in AL or AL + PQ arm. The reason why a significant Hb reduction between enrolment and day 7 occurred in heterozygous patients treated with AL alone remains unclear. Previous African safety studies have shown that heterozygous and hemizygous/homozygous malaria patients treated with higher single PQ doses (i.e., 0.4 and 0.75 mg/kg), in addition to standard ACT, were associated with statistically significant reductions in Hb on day 7, but without life threatening incidents [21, 22], whereas treatment with a lower single PQ dose, (i.e., 0.1 mg/kg) and AL alone did not result in a significant Hb reduction [22]. A study in Myanmar also revealed that, treatment with 0.25 mg/kg single-dose PQ did not result into a significant Hb reduction in G6PD deficient individuals [23]. These findings suggest that the current WHO recommendation of a single 0.25 mg/kg PQ dose is unlikely to result in life-threatening haemolysis regardless of G6PD status [4]. Of note is, however, that other factors including for example baseline parasitaemia and pre-existing anaemia have been proposed to influence the magnitude of PQ-related haemolysis in hemizygous/homozygous [6], which is in line with findings from this study.

In this study, more than half of the patients in each treatment arm recovered their Hb by day 28 to values equal to or higher than baseline, with the lowest Hb levels most often noted between days 2 and 7 after treatment initiation. This is in agreement with findings from previous studies in uncomplicated *P. falciparum* patients during follow-up after ACT treatment with or without addition of 0.75 mg/kg single PQ dose which showed that Hb dropped between days 2 and 7 and then started to recover [24–26].

A majority of the episodes of acute haemolysis in this study occurred in PCR determined G6PD wild-type/normal and heterozygous patients. Furthermore, most of the episodes of haemoglobinuria occurred in G6PD wild-type/normal patients in AL + PQ arm. Analysis of the association of pre-treatment characteristics with haemoglobinuria showed that, patients with haemoglobinuria in AL + PQ arm had significantly higher mean pre-treatment parasitaemia. These findings are in agreement with the observations that haemolysis in *P. falciparum* malaria is a common phenomenon especially in individuals with high pre-treatment parasite density [27]. Only six patients in total, three per treatment arm, fulfilled the definition of acute haemolytic anaemia. Interestingly, none were PCR determined G6PD hemizygous/homozygous, but three were phenotypically G6PD deficient. Importantly, all patients with acute haemolytic anaemia were in overall good general medical condition and recovered without medical intervention.

The phenotypic test captured 64 % of hemizygous males and 18 % of the heterozygous females. None of the homozygous females was captured by the test. However, the phenotypic test performed well in capturing wild-type/normal male and female individuals. A study in Cambodia showed a rapid test had a sensitivity and specificity of 68 and 100 %, respectively, and also it identified only 40 % of heterozygous females as deficient [28]. Conversely, in this study some patients were phenotypically deficient, but genotypically wild-type/normal. The possible explanation for this discrepancy is that, first, in this study patients with faint purple colour on the rapid tests windows were considered G6PD deficient according to the manufacture instructions, however, in a study conducted in Cambodia the faint colour was considered normal [28], thus if in this study the assessment would have been done as in Cambodia the discordance could have been reduced. Second, the genotypic test used only targeted the two most common African G6PD variants, i.e. A376G and G202A, therefore, leaving out the rare variants which were probably detected by the phenotypic test. Conversely, most heterozygous patients were missed by the phenotypic test probably because heterozygous individuals have a mixture of cells with deficient and normal G6PD activity, and the activity of the later may therefore mask the deficiency [3, 29]. However, it is not well understood why the phenotypic test missed some hemizygous and all the homozygous patients. In anaemic patients, presence of young erythrocytes masks the deficiency and may result into a false negative results by phenotypic tests [3]. However, in this study hemizygous and homozygous but phenotypically normal patients had similar baseline Hb concentration as hemizygous and phenotypically deficient patients. The herein observed and previously reported discrepancies between G6PD genotype and phenotype results underline the importance of standardization of PCR genotyping in these kinds of studies, but also calls for the development of improved phenotypic G6PD point-of-care tests. However, missing of these deficient individuals by the rapid test would pose no safety risk in the context of *P. falciparum* elimination as the findings have shown that the observed Hb drop after treatment with this single low-dose PQ was of no clinical significance. The PQ single low-dose is also recommended to be used without testing for G6PD status [6].

One of the strength of this study is that patients were included regardless of G6PD status and randomly allocated to treatment with or without a single low-dose of PQ in addition to standard AL regimen. Furthermore, this is one of the few studies to look at the 0.25 mg/kg dose in Africa and contributes to an evidence base that was otherwise lacking. Limitations of the study include: that some young children had difficulties to remember and explain potential adverse events. Only the two most common African G6PD deficient A376G and G202A variants were genotyped, hence potentially missing the rare variants which could have explained the discrepancies observed between phenotypic and genotypic tests. No further assessment was done to confirm whether the observed Hb drop was due to other factors than haemolysis. Only patients with Hb ≥8 g/dL were included in the study, hence it is difficult to predict how patients with lower Hb would respond to PQ treatment particularly at the community level where the impact of PQ use is of more significant. However, asymptomatic parasites carriers are the target at the community level, and usually this group has low parasitaemia and Hb level that does not pose a risk with PQ use, particularly with this single low-dose as recommended by WHO.

## Conclusion

The findings from this study support the new WHO recommendation that the addition of a single low-dose of PQ (0.25 mg/kg) to standard AL is safe for the treatment of uncomplicated acute *P. falciparum* malaria in Tanzania regardless of individual G6PD status.

## Additional file

10.1186/s12936-016-1341-3 Characteristics of patients with acute haemolytic anaemia during the trial.

## Abbreviations

ACT: artemisinin-based combination therapy
AL: artemether-lumefantrine
AE: adverse event
CI: confidence interval
DNA: deoxyribonucleic acid
GCP: good clinical practices
G6PD: glucose-6-phosphate dehydrogenase deficiency
Hb: haemoglobin
LLIN: long-lasting insecticide-treated mosquito nets
N: sample size
NADP: nicotinamade adenide dinucleotide phosphate
NMCP: national malaria control programme
PCR: polymerase chain reaction
PQ: primaquine
PROMPT: primaquine roll out monitoring pharmacovigilance tool
RFLP: restriction fragment length polymorphism
SAE: serious adverse event
SD: standard deviation
SPSS: statistical package for social sciences
WBC: white blood cell
WHO: World Health Organization
