# Supplementary material for: Safety of a single low-dose of primaquine in addition to standard artemether-lumefantrine regimen for treatment of acute uncomplicated Plasmodium falciparum malaria in Tanzania
Source: Malar J. 2016 Jun 10;15:316. doi: 10.1186/s12936-016-1341-3 (PMC4901409; doi:10.1186/s12936-016-1341-3)
Supplement: Supplementary file 1 — 10.1186/s12936-016-1341-3 Characteristics of patients with acute haemolytic anaemia during the trial. [file 12936_2016_1341_MOESM1_ESM.doc]

**Additional file 1: Characteristics of patients with acute ha**emolytic anaemia during the trial.

| **Characteristic** | **Patient** | | | | | |
| --- | --- | --- | --- | --- | --- | --- |
|  | 1 | 2 | 3 | 4 | 5 | 6 |
| Age (years) | 23 | 3 | 30 | 2 | 4 | 1 |
| Sex  Baseline parasitaemia/µL  Phenotypic G6PD status  Genotypic G6PD status | Female  275880  Deficient  Normal | Male  87200  Normal  Normal | Female  8280  Normal  Heterozygous | Female  44240  Normal  Normal | Female  196160  Deficient  Normal | Male  168040  Deficient  Normal |
| Day 0 Hb (g/dL) | 8.7 | 10.6 | 13.5 | 8.8 | 8.2 | 8.1 |
| Day 7 Hb (g/dL) | 8.6 | 8.6 | 10.1 | 6 | 6.8 | 6.6 |
| Day 28 Hb (g/dL)  Day of Hb nadir | 11.7  2 | 10.4  2 | 12.1  10 | 10.9  7 | 11.1  2 | 10.9  2 |
| Treatment arm | AL | AL+PQ | AL+PQ | AL | AL+PQ | AL |
